# The long-term associations between parental behaviors, cognitive function and brain activation in adolescence

**DOI:** 10.1038/s41598-021-90474-2

**Published:** 2021-05-27

**Authors:** Orwa Dandash, Nicolas Cherbuin, Orli Schwartz, Nicholas B. Allen, Sarah Whittle

**Affiliations:** 1 Melbourne Neuropsychiatry Centre, Department of Psychiatry, University of Melbourne, Level 3, Alan Gilbert Building, 161 Barry St, Carlton, VIC 3053 Australia; 2 Centre for Research on Ageing, Health and Wellbeing, ANU College of Health and Medicine, Acton, ACT Australia; 3 Orygen, Centre for Youth Mental Health, University of Melbourne, Parkville, VIC Australia; 4 Department of Psychology, The University of Oregon, Eugene, OR USA

**Keywords:** Cognitive control, Cognitive neuroscience, Development of the nervous system, Stress and resilience

## Abstract

Parenting behavior has a vital role in the development of the brain and cognitive abilities of offspring throughout childhood and adolescence. While positive and aggressive parenting behavior have been suggested to impact neurobiology in the form of abnormal brain activation in adolescents, little work has investigated the links between parenting behavior and the neurobiological correlates of cognitive performance during this age period. In the current longitudinal fMRI study, associations between parenting behaviors and cognitive performance and brain activation across mid- and late-adolescence were assessed. Observed measures of maternal aggressive and positive behavior were recorded in early adolescence (12 years) and correlated with fMRI activation and in-scanner behavioral scores on the multi-source interference task (MSIT) during mid- (16 years; 95 participants) and late-adolescence (19 years; 75 participants). There was a significant reduction in inhibitory-control-related brain activation in posterior parietal and cingulate cortices as participants transitioned from mid- to late-adolescence. Positive maternal behavior in early-adolescence was associated with lower activation in the left parietal and DLPFC during the MSIT in mid-adolescence, whereas maternal aggressive behavior was associated with longer reaction time to incongruent trials in late-adolescence. The study supports the notion that maternal behavior may influence subsequent neurocognitive development during adolescence.

## Introduction

Adolescence is an important developmental stage during which the brain matures and undergoes significant structural and functional changes^1^. The frontal lobe in particular plays a pivotal role in orchestrating higher cognitive functions that include planning, attention, working memory and impulse inhibition^2,3^. The protracted development of the frontal lobe during adolescence can be beneficial in terms of convention learning, including language and social norms^4^, but may also potentiate the effect of negative social environmental factors that can have long-term effect on brain development and cognitive abilities^5^.

Cognitive abilities such as interference resolution that enables the individual to focus on task related information is governed by a host of factors including not only intelligence but also affective attributes such as motivation and impulse control^6^. Early life stressors have been shown to delay brain maturation and to lead to impaired self-control in early adolescence^7^. These impairments may in turn predispose the individual to risk-taking and impulsive behavior associated with reduced attention and behavioral disorders such as gambling and substance abuse^8^. Furthermore, individual differences in self-control predict academic achievement, risk-taking, criminal behavior and income in young people regardless of their intelligence and socioeconomic status^9^.

One particularly important set of early life stressors that has been associated with alterations to healthy brain development is parental behaviors and interactions with children. The emotional state of the parent as well as their interaction with their child has been associated with altered brain structure and function, and cognitive function in children and adolescents^10–13^. Regions of the cingulo-fronto-parietal (CFP) brain network have been particularly implicated. This network is known for its role in response inhibition and cognitive function^14^. Accumulating evidence shows that the CFP network activates during tasks that require flexible control of goal-directed behavior as well as when adjusting cognitive responses to environmental cues^15^, behavioral constructs that are reduced in youth exposed to aversive parental behavior^16^. Conversely, adolescents who receive higher-levels of parental warmth show better academic performance and inhibitory control during childhood^17,18^. Previous work with the current sample has shown that maternal aggression (i.e., expression of anger, contempt and belligerent or provocative attitude) during an event planning interaction (EPI) with their adolescent child was associated with reduced cortical thinning in the CFP brain network involving frontal and parietal regions across adolescence^11^. These structural changes were predictive of functional outcomes such as school performance and academic achievement in adolescence^11^. Conversely, low levels of maternal positive behaviors (including happy and caring affect as well as approving or validating comments) during a problem solving interaction (PSI) were associated with accelerated cortical thinning in frontal regions^11,12^.

In this study we take these findings further by investigating the neurobiological mechanism that are believed to underlie the association between maternal behavior, brain development and cognitive function in adolescence using a longitudinal design. To our knowledge, this is the first longitudinal, large cohort fMRI study that aims to examine the prospective association between maternal behavior during early adolescence and developmental changes in neurocognitive function across mid- and late-adolescence. To this end we utilized the Multi-source Interference Task (MSIT), a block-design cognitive fMRI task that reliably activates cognitive and attention brain networks including the canonical CFP network^14,19^. The MSIT targets an important aspect of cognition that draws into multiple cognitive domains including attention, interference resolution and inhibition, cognitive constructs that have been shown to be affected by parental behavior^20^.

Previous work with the MSIT provides support for normative changes across adolescence; for example, the interference effect of the MSIT has been found to correlate with increased activation in the prefrontal cortex^21^ followed by a reduction in activation with advancing age in healthy adolescent subjects^22^. Other work has shown that behavioral issues linked to both parenting behaviors^23^ and cognition^24^, are associated with alterations in MSIT-related brain activation. For example, in a group of healthy adolescents, externalizing behavior predicted increased activation of the parietal lobe while internalizing behavior predicted activation in the prefrontal cortex during the MSIT^25^. These results support the utility of the MSIT in investigating links between parenting behaviors and neurobiological correlates of adolescent cognitive function.

The study aimed to investigate the prospective association between maternal behavior during parent child interactions during early adolescence and patterns of neurocognitive development across mid- to late-adolescence. To this end, mother-adolescent interactions on the EPI and the PSI were recorded and maternal behavior from these interactions was correlated with longitudinal fMRI activation during performance of the MSIT. Based on the limited literature in which MSIT has been utilized to assess cognitive function in healthy adolescent subjects, and the broader literature assessing longitudinal changes in cognitive function in relation to early maternal behavior, we predicted a) reduced activation of the CFP cognitive brain network in a manner similar to the adult brain with advancing age, b) aggressive maternal behavior during an EPI to be associated with poorer cognitive performance on the MSIT and with age-related increased activation in the parietal lobe, and c) positive maternal behavior during a PSI to be associated with better performance on the MSIT and age-related reduction in brain activation in the CFP.

## Results

**Figure 1:**
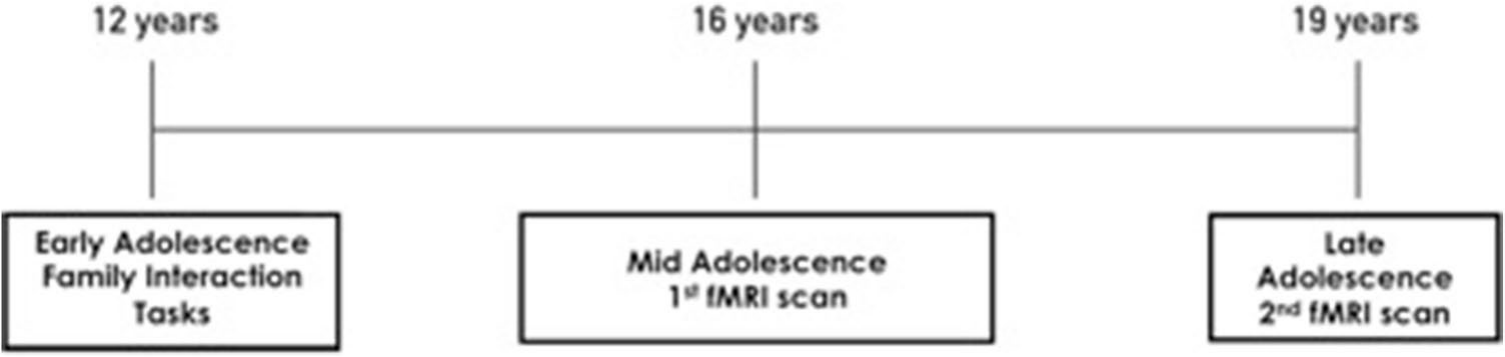
Study timeline.

A final sample of 129 participants in mid-adolescence (47% males; Age 16.47 (0.53) years) and 99 participants in late-adolescence (47% males; Age 18.81 (0.47) years) were included in the analysis of this report as shown in Table 1. Of these participants parenting data in early adolescence was available for 95 participants in mid adolescence (mother’s aggressive behavior in the EPI: M 0.66 SD 0.46; mother’s positive behavior in the PSI: M 2.26, SD 0.53) and 75 participants in late adolescence (mother’s aggressive behavior in EPI: M 0.75, SD 0.43; mother’s positive behavior in PSI: M 2.26, SD 0.48; Table 1). There was no difference between this sample and the original sample of 245 participants that entered the study initially by baseline age, sex, or SES (*p* > 0.05) (Fig. 1).

**Table 1:** Demographics and characteristics of the sample. Ethnicity was assessed based on genetics data for carrying the highest percentage of African, Asian or Caucasian alleles. SES: Socioeconomic Status, indicated by parental occupation status and assessed based on the Australian National University Four Scale^26^. FSIQ: Full-scale IQ test was assessed using a short form of the Wechsler Intelligence Scale for Children, 4th Ed. Maternal behavior was recorded in early-adolescence.

Characteristics	Sample (N = 129)
Ethnicity	92% Caucasian 8% Asian
Age (Mid-adolescence) Mean (SD)	16.47 (0.53)
Age (Late adolescence) Mean (SD)	18.81 (0.47)
Sex-16 years (M/F)	61/68
Sex-19 years (M/F)	47/52
SES Mean (SD)	58.00 (20.30)
FSIQ Mean (SD)	105.61 (12.63)
Mother’s aggressive behavior in EPI Mean (SD)	0.49 (0.46)
Mother’s positive behavior in PSI Mean (SD)	1.74 (0.68)

### fMRI activation & mapping

**Figure 2:**
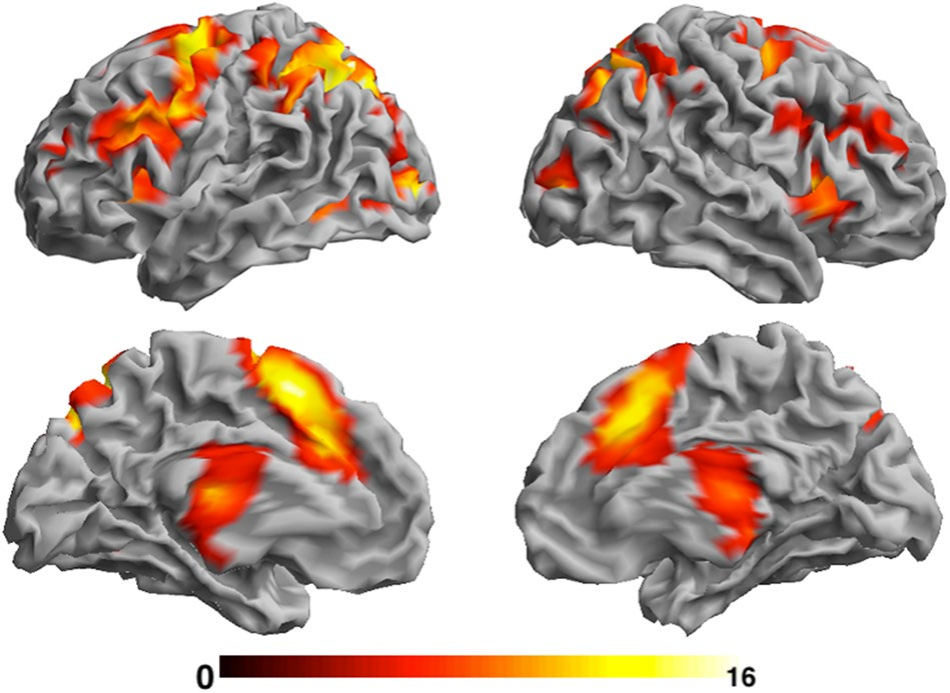
Statistical parametric map showing main MSIT activation effect (incongruent > congruent) across both time points. The colour bar represents standardised Z-scores. Right hemispheres are shown on the right. Results are corrected for multiple comparisons (*p* < .05; see Methods” for more details).

A significant MSIT activation effect (brain activation to the Incongruent > Congruent contrast) was detected in brain regions representing the CFP brain network; the dorsomedial and ventromedial prefrontal cortex, dorsolateral prefrontal cortex, posterior parietal cortex and thalamus across both time points (Fig. 2; Supplementary Table S1) consistent with our hypothesis that MSIT can reliably activate the CFP brain network and with previous findings^14,27^.

### Temporal differences in MSIT activation effect

**Figure 3:**
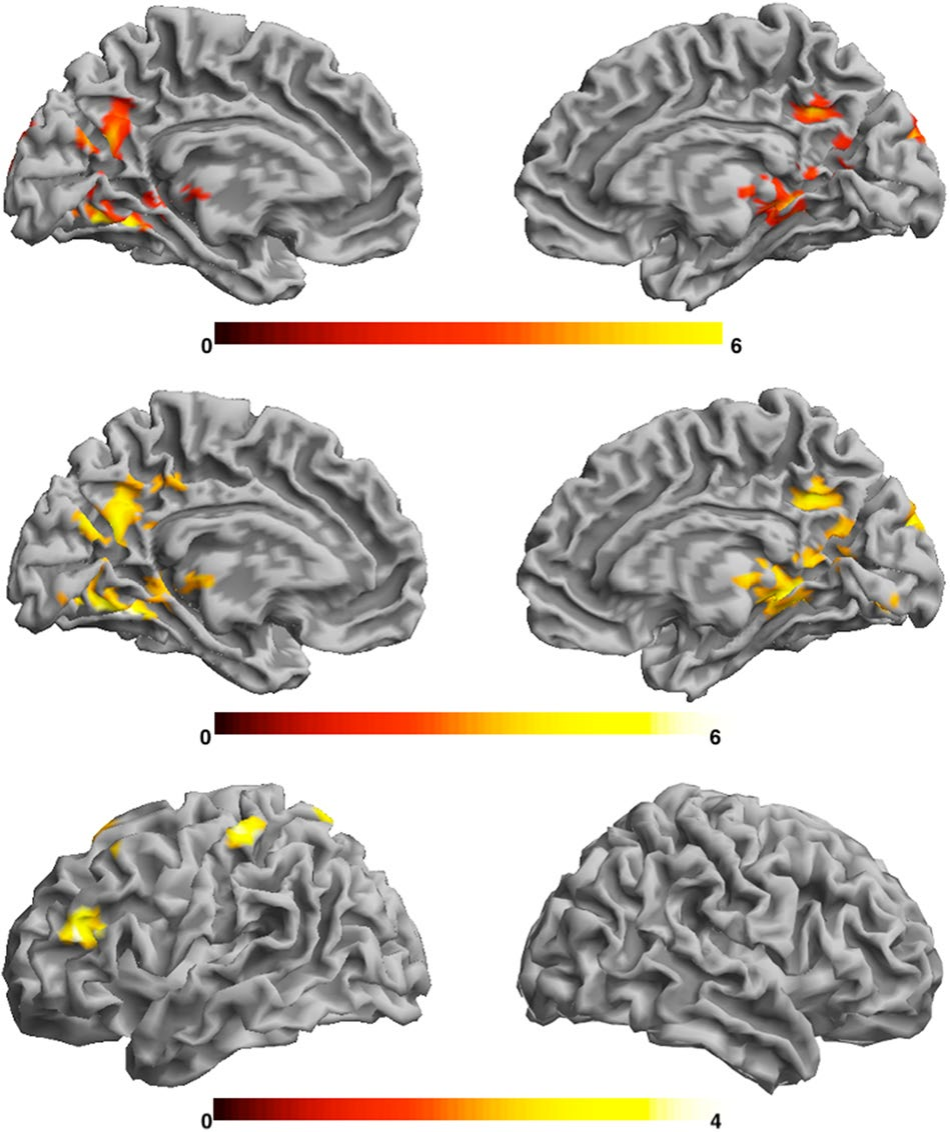
Statistical parametric map showing the time x condition interaction (top), the confirmatory t-test results of temporal differences in brain activation (mid-adolescence > late-adolescence) in the MSIT activation effect (middle) and negative association between MSIT activation effect in mid-adolescence and maternal positive behaviour in early-adolescence (bottom). Colour bars represent standardized Z-scores. Right hemispheres are shown on the right. Results are corrected for multiple comparisons (*p* < .05; see “Methods” for more details).

This test showed activation changes in the MSIT activation effect (Incongruent > Congruent contrast) over time. The Incongruent > Congruent contrast was greater in mid-adolescence compared to late adolescence, suggesting greater differences in brain activity between these conditions during mid-adolescence when compared to late-adolescence. The effect was detected in the posterior cingulate cortex, posterior parietal and visual cortex (Fig. 3; Supplementary Table S1). Imaging results remained significant with participant sex included as a variable of no interest in the model.

### In-scanner behavior analysis

**Figure 4:**
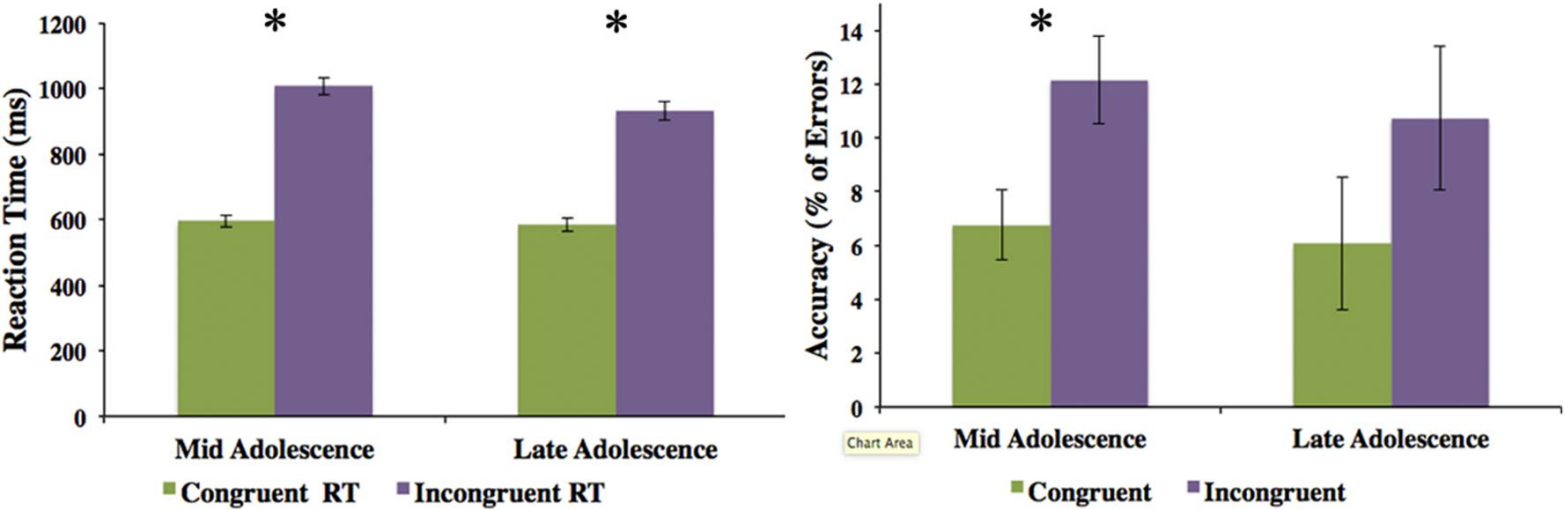
Differences in Reaction Time (ms) and Accuracy between congruent and incongruent trials in mid-adolescence and late-adolescence. Accuracy was calculated as the percentage of commission errors made to each corresponding trial. Bars represent 95% CI, (*p < .0001). See Table 2 for more information.

**Figure 5:**
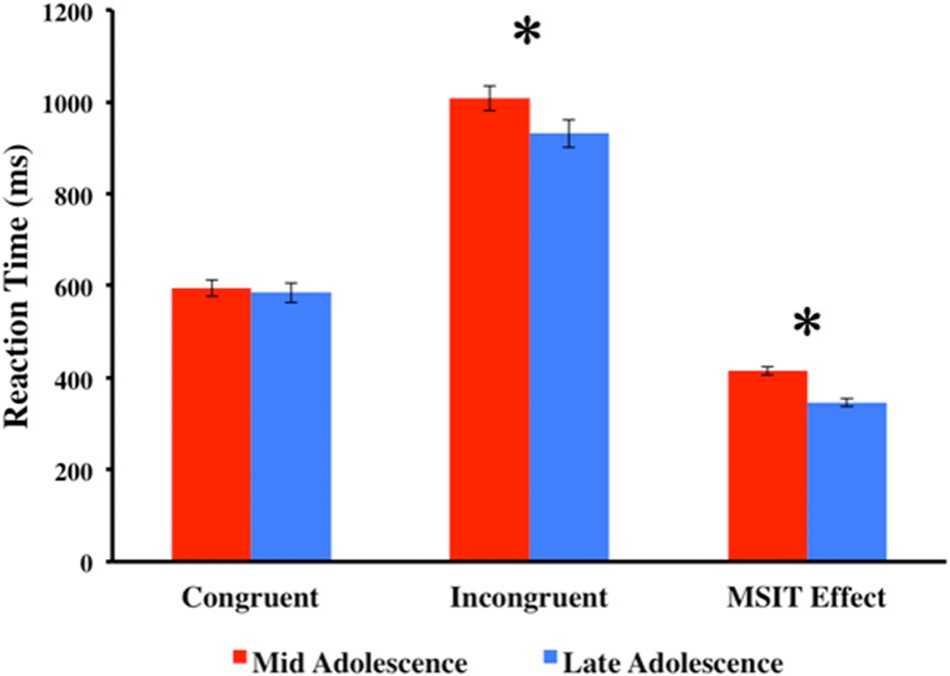
Differences in Reaction Time (ms) between congruent and incongruent trials across mid-adolescence and late-adolescence. MSIT behavioural effect was calculated by subtracting reaction time of congruent trials from reaction time of incongruent trials of each time point (*p < .001). Bars represent 95% CI. See Table 3 for more information.

There was a significant MSIT behavioral effect (i.e., difference in reaction time) between congruent and incongruent trials in mid- and late-adolescence (Fig. 4; Table 2). Participants responded faster and more accurately to congruent trials than to incongruent trials at both time points (Fig. 4; Table 2). Longitudinally, there was a significant difference in reaction time to incongruent trials only between the two time points, reflecting that participants responded more quickly to incongruent trials in late adolescence compared to mid-adolescence (Fig. 5; Table 3). Accuracy did not differ between the two time points for either the congruent or incongruent trials (Table 3).

**Table 2:** Reaction time (RT) and accuracy scores for the congruent and incongruent trials in mid-adolescence and late-adolescence. See Fig. 4 for more information.

Scan	Congr. RT	Incong. RT	t-test (df)	p	Cong % error (Accur.) Mean (SD)	Incong % error (Accur.) Mean (SD)	t-test (df)	p
Mid Adolesc	594.25 (174.79)	1007.48 (236.74)	−25.36 (270)	.0001	6.76 (7.74)	12.15 (9.76)	5.051 (270)	.0001
Late Adolesc	584.46 (147.35)	931.07 (199.75)	−18.65 (182)	.0001	6.07 (12.10)	10.73 (13.11)	2.508 (182)	0.013

**Table 3:** Longitudinal comparison of reaction time and accuracy between mid-adolescence and late-adolescence. See Fig. 5 for more information.

Trials	Mid Adolesc RT Mean (SD)	Late Adolesc RT Mean (SD)	t-test (df)	p	Mid Adolesc Accur. Mean (SD)	Late Adolesc Accur. Mean (SD)	t-test (df)	p	MSIT Behavior Effect Mid Adolesc	MSIT Behavior Effect Late Adolesc	t-test	p
Cong	594.25 (174.79)	584.46 (147.35)	.701 (226)	.484	6.76 (7.74)	6.07 (12.10)	-.523 (226)	.602	415.85 (106.03)	346.61 (85.96)	5.21 (226)	.0001
Incong	1007.48 (236.74)	931.07 (199.75)	3.68 (226)	.0001	12.15 (9.76)	10.73 (13.11)	-.933 (226)	.352				

### Maternal behavior and neuroimaging

The linear regression models showed that maternal aggressive and positive behaviors during early adolescence were not associated with developmental change in activation for the Incongruent > Congruent contrast in the MSIT detected over time. However, a follow up exploratory analysis demonstrated an association between higher frequency of positive maternal behavior and lower activation in the CFP (left DLPFC and posterior parietal cortex) in mid-adolescence (Fig. 3; Supplementary Table S1).

Mother’s aggressive behavior was associated with longer reaction time to incongruent trials in late-adolescence such that the higher the frequency of the expressed aggressive maternal behavior the longer it took the child to respond to the incongruent trials (p = 0.022, Dof = 75, r = 0.265). No other associations between maternal behavior during early adolescence and measures of reaction time or accuracy at either time point were detected.

## Discussion

The results showed significant reduction in activation in posterior cingulate and parietal cortex during the progression from mid- to late-adolescence on the MSIT. Participants also demonstrated higher control effect by responding more quickly to incongruent trials in late adolescence than in mid-adolescences, but with no change in accuracy. In addition, maternal positive behavior in early-adolescence negatively correlated with activation to the MSIT in the left parietal cortex and DLPFC, while maternal aggressive behavior was associated with longer reaction time to incongruent trials in late-adolescence.

The findings of developmental changes in brain activity and performance during the MSIT are consistent with prior fMRI studies in typically developing children and adolescent subjects^22^. This prior work has shown that both response time and accuracy increase as the age of the subject increases from 8 to 19 years. Neurodevelopmental research has attributed these effects to the observed maturation and strengthening in the neural circuitry that supports cognitive function in the adolescent brain^28,29^. This includes rewiring and pruning of redundant white matter connections especially in the frontal cortex that promote efficient cognitive activity and enhanced cognitive abilities^1^. In particular, the maturation of the cingulo-fronto-parietal (CFP) network that is believed to form the core of human task-set system that mediates the consolidation of high order executive function involving attention and response inhibition required to achieve better task performance^30^. Hence, the longitudinal reduction in activation coupled with faster response time and no change in accuracy may reflect age related maturation in response to cognitive demands that aim at consolidating a more efficient executive function.

While maternal behavior did not predict a change in brain activation over time it showed significant association with activation in the CFP brain network in mid-adolescence. Specifically, maternal positive behavior was associated with lower activation in the left DLPFC and parietal regions in mid-adolescence. Abnormally increased activation in response to cognitive stimuli in the DLPFC and parietal areas has been detected in patients with anxiety and other mental disorders^31^ whereas children exposed to childhood trauma demonstrate heightened activation later in life^32^. It is possible that adolescents that experienced more positive maternal behaviors were more confident in their choices and had more efficient executive processing due to better inhibition control and attention than adolescents exposed to lower levels of maternal positivity. In early adolescents, maternal warmth and support has been shown to be significant negatively correlated with adolescent brain activation during exposure to negative affective stimuli^33^. The opposite effect has been detected in maltreated adolescents^34^. Together, these findings suggest that positive maternal behavior in early adolescence may contribute to the long-term normalizing of brain activation by having an enduring effect on brain structural integrity and function.

A notable feature of this study is the importance of mid-adolescence in the development of the neural circuitry involved in cognitive function. This was demonstrated in the increased activation of posterior brain regions (Fig. 3; Supplementary Table S1) that normalizes in late-adolescence. Mid-adolescence is a crucial period in which most adolescents have undergone puberty but remain in the transitioning stage between childhood and early adulthood. It is a time with heightened sense of independence as well as increased onset of mental disorders^35^. Hence, social environmental factors such as parental interactions, may have their highest effect in this period of time^12,36^. Although parental factors in childhood have been shown to correlate with brain activation and structural changes decades later^32^, our study supports the role of maternal behavior during the adolescent years on cognitive function of adolescents in this critical period of human development.

The study was limited in size due to the inclusion of participants for whom both behavioral and imaging data were available for the two time points under investigation; mid- and late-adolescence. Only maternal-adolescent interaction was investigated in this study due to the fact that the vast majority of participating parents/guardians were mothers. Hence, extrapolation of the results to paternal aggressive and positive behavior requires further investigation. In addition, the small variability in the mother’s aggressive behavior, compared to positive behavior, may have limited our ability to detect effects. It is also important to acknowledge that laboratory-based interactions likely differ from those that occur in day-to-day interactions. Nevertheless, laboratory-based family interactions have good predictive and convergent validity with other measures of these processes as well as with depressive syndromes, suggesting that they capture valid and important information regarding family interactions^37,38^.

The relatively large sample size and the longitudinal nature of the study design are all strengths that provide better assessment of temporal changes in neurobiological changes that are lacking in cross-sectional studies. In conclusion, the study supports a role for maternal behavior in predicting adolescent cognitive function possibly through changes in neurobiology.

## Methods

The study was approved by the Human Research Ethics Committee of The University of Melbourne and all experiments were performed in accordance with the regulations and guidelines endorsed by the Committee. The data were collected as part of the Orygen Adolescent Development Study (ADS), a longitudinal research project that has been described in full details elsewhere^3,13,39^. Briefly, the ADS investigated social, psychological and biological risk factors for psychopathology from 12–19 years of age in participants from the local community in metropolitan Melbourne, Australia. Participants were initially included to have varying levels of temperamental risk for psychopathology. At the commencement of the study, 2479 6th grade participants were screened using the Early Adolescent Temperament Questionnaire–Revised (EATQ-R) and 245 selected participants agreed to participate in the study. Participants were excluded if there was any evidence of current or previous depressive, substance use, or eating disorder. They were also excluded from neuroimaging if there was evidence of chronic illness, language or learning disabilities, or use of medicines known to affect nervous system functioning.

The present study used mother–adolescent interaction data available from the early-adolescence (12 years old) assessment, and task functional magnetic resonance imaging (fMRI) data from assessments when participants were approximately 16 and 19 years old. Written informed consent was obtained for all participants and their parent or guardian before their inclusion in the study in accordance with the human research ethics committee of The University of Melbourne.

### Initial diagnostic screening

The Schedule for Affective Disorders and Schizophrenia for School-Aged Children: Present and Lifetime Version^40^ was administered at screening to assess for lifetime diagnoses of DSM-IV Axis I disorder. Interviews were conducted by trained researchers under the supervision of the principal investigator. This is in addition to a clinical psychologist who met with researchers once a week to discuss symptoms and diagnoses. Approximately 20% of interviews were double-scored by another researcher, and inter-rater reliability was calculated on all items, including symptoms and diagnoses, (Excellent range Kappa of > 0.77^40^).

### Adolescent–mother interactions

Due to the very small number of male parents attending interviews (N = 35) the study was limited to female parents. Participants and their mothers participated in two 20-min family interactions: an event-planning interaction (EPI) followed by a problem-solving interaction (PSI). The topics of the two tasks were identified based on participant responses to the Pleasant Events Checklist and the Issues Checklist^41^, respectively, and were video-recorded for coding purposes. The Pleasant Events Checklist is composed of activities that people enjoy doing, whereas the Issues Checklist contains topics with the potential to create mother-adolescent conflict. The Living In Family Environments (LIFE) coding system^42^ was used to obtain a detailed analysis of mothers’ and children’s behaviors from the video of the interactions. The LIFE consists of 10 nonverbal affect codes and 27 verbal content codes. Coding of interactions used an event-based protocol in which new codes were entered each time the affect or content of one of the interactants changed. The affect and verbal content codes were used to develop composite positive and aggressive expression constructs. The positive construct included all behaviors with happy or caring affect and approving, validating, affectionate, or humorous comments made with neutral affect. The aggressive construct included all behaviors with contemptuous, angry, or belligerent affect and cruel, provocative, annoying or disruptive, or argumentative verbal statements made with neutral affect. LIFE data were used to construct a measurement of behavioral frequency for each construct, calculated as the rate per minute of a particular behavioral expression (i.e., the average number of times a mother expressed a behavior type [i.e., aggressive or positive] per minute). We have previously found that maternal expression of aggressive and positive behavior that is “out of context” (i.e., aggressive behavior expressed during a positive interaction task, low levels of positive behavior expressed during a negative task) prospectively predicts the onset of adolescent mental health problems^43^, suggesting that such behaviors may be particularly maladaptive. As such, maternal aggression during the EPI and positive behavior during the PSI were investigated in the current study. Similar to the clinical interview, approximately 20% of LIFE interviews were double-scored by another researcher, and inter-rater reliability was calculated on all items, (Excellent range Kappa of > 0.77^40^).

### The MSIT fMRI task

Each participant performed the Multi-Source Interference Task (MSIT), a block-design fMRI paradigm designed to assess interference control, or behavioral inhibition, which reliably activates the cingulo-fronto-parietal (CFP) cognitive brain network at a single-participant level (Bush and Shin, 2006). The task was administered using Presentation software (Neurobs; v0.70) and viewed via a head-coil mirror on a screen that was placed at the participant’s feet. A button pad with three-button press was provided to participants so that their index, middle, and ring fingers corresponded to the numbers 1, 2, and 3, on the button pad, respectively. They were then shown a row of three numbers, two of which were matching (e.g., “1 2 2”), and they were asked to press the button that corresponded to the unmatched number. During congruent trials, the unmatched number was placed in the same position as the finger response. For example in the previous example the unmatched number place corresponded to the index finger button press, while in interference trials the unmatched number was moved to other locations to induce finger-to-number incongruence (e.g., “3 3 1”). The task was organized in a block design with eight alternating blocks of 30 s of congruent and incongruent trials. Each trial lasted for 2 s and had an intertrial interval of 500 ms. Blocks of 30 s visual fixation were interleaved between the congruent and incongruent blocks. Task performance was measured using MSIT interference reaction time (RT) score (MSIT behavioral effect), calculated by computing the difference between incongruent and congruent trials whereby lower scores indicated less interference while higher scores indicated more interference. MSIT behavioural response accuracy was also calculated for each participant by summing the commission errors (pressing the wrong button) during both congruent and incongruent trials. Trials with omission errors were excluded from the analysis. For descriptive reporting of accuracy the scores were also represented as a percentage of trials overall.

### Neuroimaging acquisition and preprocessing

MRI was performed for each participant in a single session with a 3 T Siemens Magnetom Trio B15 whole-body scanner at the Royal Children’s Hospital, Victoria, Australia as has been described in details elsewhere^3,13^. Participants’ heads were immobilized using foam padding and a 32-channel head coil. Task fMRI scanning was performed using single-shot gradient-recalled EPI providing T2*-weighted BOLD contrast with the following parameters: TR 2400 ms, TE 40 ms, flip angle 90°, FOV 210 mm, voxel size 3.3 × 3.3 × 3.0 mm, 36 slices, 157 whole-brain volumes. Structural images were acquired as a gradient echo volumetric acquisition sequence (TR 1900 ms, TE 2.24 ms, FOV 230 mm) to obtain 176 T1-weighted contiguous 0.9 mm slices (0.9 × 0.9 × 0.9 mm).

Image preprocessing was performed using Statistical Parametric Mapping (SPM8; http://www.fil.ion.ucl.ac.uk/spm/) software and included motion correction by affine transformation to the first image and co-registration of functional images with participants’ anatomic scans, which were concurrently normalized to the SPM-T1 template. One hundred and forty participants undertook the Multi-source Interference Task (MSIT) in the first (mid-adolescence) MRI scan whereas 107 participants participated in the second (late adolescence) MRI scan (Fig. 1). Four participants in mid-adolescence and two participants in late-adolescence were excluded due to poor scan coverage. In addition, seven participants in mid-adolescence and six participants in late-adolescence were excluded due to head motion exceeding 3 mm translation or 3 degrees rotation.

The resulting transformation matrix was applied to the functional data to achieve accurate spatial normalization across individuals. The anatomic scans were segmented using a unified normalization and segmentation approach^44^. The SPM intensity-based segmentation algorithm thresholding values for cerebrospinal fluid (CSF) and white matter (WM) were selected to create minimal overlap between the different segmented tissue types while ensuring that gray matter tissues are not overly penalized. WM and CSF segments were generated by thresholding the corresponding tissue images segmented from the T1 scan at 99% and 50% tissue probability, respectively. The resultant combined WM and CSF mask was subtracted from the gray matter mask with a stringent threshold of 1% so that voxels containing more than 1% WM or CSF were removed. The final WM plus CSF mask was used to extract spurious signal from the data.

To further control for noise and spurious signal, voxel-wise time series were extracted from the WM and CSF masks and subjected to principal component analyses using a CompCor method^45^. The first 5 components were retained from each analysis. A linear regression model that included these 10 component signals and the 6 head motion parameters (3 rotation, 3 translation) estimated during the head motion correction procedure and the first-order derivatives of all 16 signals were fitted on a voxel-wise basis^46^. The noise-corrected data were high-pass filtered (f > 0.08) and spatially smoothed with a Gaussian filter (8 mm full-width at half-maximum). All image sequences were routinely inspected for potential normalization artifacts.

### First-level modeling

The general linear model (GLM), as implemented in SPM8 was used to conduct a whole-brain voxel-wise analysis to define the task-activated cingulo-fronto-parietal (CFP) brain network. The blocks from each of the three conditions of the task (fixation, congruent, and incongruent) for each time point were coded as individual regressors, convolved with a hemodynamic response function (box car) and incorporated as covariates in a GLM that was fitted on a voxelwise basis to the measured BOLD signal. For each participant, the CFP brain network was defined as regions showing statistically significant activation during incongruent trials compared with congruent trials (Bush and Shin 2006).

### Second-level modeling

A second-level random-effects full factorial model was built to examine the main effect of both variables (time and condition) as well as the interaction between them. Confirmatory *t* tests followed to examine differences in the MSIT activation effect over time. To this end, the t-test results were masked by the interaction results to ensure that detected effects are limited to the interaction term. Model parameters were estimated using Restricted Maximum Likelihood (ReML). To investigate associations with maternal behavior, frequencies of aggressive and positive maternal expression were entered as covariates of interest in separate models. Similarly, reaction time and accuracy scores were entered into separate models to investigate associations with activation in the CFP brain network. Group-level statistical maps were subjected to initial voxel-wise thresholding of *p* < 0.001 for viewing purposes and then were subjected to cluster-based family-wise error correction (*p* < 0.001) using Gaussian random field theory as implemented in SPM to correct for multiple comparisons. Statistical maps were further tested for significance using a nonparametric, threshold-free cluster enhancement method^47^ at a threshold of *p* < 0.05, and only maps that survived both correction methods are reported herein.

## Supplementary Information

Supplementary Information.

## Data Availability

The datasets generated during and/or analysed during the current study are available from the corresponding author on reasonable request.
