# Supplementary material for: The long-term associations between parental behaviors, cognitive function and brain activation in adolescence
Source: Sci Rep. 2021 May 27;11:11120. doi: 10.1038/s41598-021-90474-2 (PMC8160361; doi:10.1038/s41598-021-90474-2)
Supplement: Supplementary file 1 — Supplementary Information. [file 41598_2021_90474_MOESM1_ESM.doc]

**The long-term associations between parental behaviors, cognitive function and brain activation in adolescence**

Orwa Dandash^1,2*^, Nicolas Cherbuin^2^, Orli Schwartz^3,4^, Nicholas B. Allen^5^, and Sarah Whittle^1^

**Supplementary Table S1:**

Brain regions demonstrating significant activation to the MSIT across both time points and association with mother’s aversive behavior in mid-adolescence (*P*<.05 FWE Cluster Corrected).

| **Main Effect** | **Anatomical Region** | **Hemisphere** | **MNI Peak Coordinates (x y z)** | **Z-score** | **Cluster-size (Voxels)** |
| --- | --- | --- | --- | --- | --- |
| **MSIT** | Posterior Parietal Cortex | Left | -24 -70 46 | 16.10 | 38998 |
|  | Medial PFC | Left | -6 10 52 | 14.96 |  |
|  | Premotor Cortex | Left | -30 -6 62 | 13.94 |  |
|  | DLPFC | Left | -44 2 32 | 12.92 |  |
|  | Visual Cortex | Left | -30 -94 0 | 12.71 |  |
|  | VLPFC | Right | 36 18 10 | 12.38 |  |
|  | VLPFC | Left | -32 22 12 | 10.97 |  |
|  | Posterior Parietal Cortex | Right | 28 -66 50 | 10.67 |  |
|  | Thalamus | Left | -8 22 32 | 10.62 |  |
| **Condition**  **X**  **Time Interaction** | Medial Temporal Cortex | Left | -30 -62 -10 | 5.78 | 1465 |
|  | Visual Cortex | Right | 26 -84 20 | 4.91 | 495 |
|  | Posterior Cingulate Cortex | Right | 14 -50 36 | 4.39 | 1237 |
|  | Posterior Insula | Left | -36 -26 4 | 4.21 | 200 |
| **Behavioural Association** | DLPFC | Left | -44 32 18 | 4.12 | 268 |
|  | Posterior Parietal Cortex | Left | -50 -30 60 | 3.90 | 153 |
